# Text message-based lifestyle intervention in primary care patients with hypertension: a randomized controlled pilot trial

**DOI:** 10.1080/02813432.2020.1794392

**Published:** 2020-07-20

**Authors:** Beata Borgström Bolmsjö, Moa Wolff, Veronica Milos Nymberg, Magnus Sandberg, Patrik Midlöv, Susanna Calling

**Affiliations:** a Department of Clinical Sciences Malmö, Lund University, Sweden; b Center for Primary Healthcare Research, Skåne University Hospital, Lund University, Sweden; c Department of Health Sciences, Lund University, Sweden

**Keywords:** Primary health care, hypertension, RCT, feasibility study, reminder systems, mobile phones, lifestyle advice

## Abstract

**Objective:**

To evaluate the feasibility of a randomized controlled pilot study with lifestyle-promoting text messages as a treatment for hypertension in primary care.

**Design:**

Randomized controlled pilot trial.

**Setting:**

Three primary health care centers in southern Sweden.

**Subjects:**

Sixty patients aged 40–80 years with hypertension were included.

**Main outcome measures:**

Feasibility of the pilot study, i.e. recruitment rate, dropout rate and eligibility criteria. Secondary outcomes were change in blood pressure and other cardiovascular risk factors.

**Methods:**

Thirty participants were randomized to the intervention group with four lifestyle-promoting text messages sent every week for six months. The control group received usual care. The baseline and follow-up visits for all 60 patients included measurements of blood pressure, anthropometrics, blood tests and a self-reported questionnaire.

**Results:**

All feasibility criteria (recruitment rate (≥55%), dropout rate (≤15%) and eligibility (60 eligible patients during the four-month inclusion period) for the pilot study were fulfilled. This means that a larger study with a similar design may be conducted. After six months, there were no significant improvements in cardiovascular risk factors. However, we found favorable trends for all secondary outcomes in the intervention group as compared to the control group.

**Conclusion:**

Lifestyle modification in patients with hypertension is important to reduce cardiovascular risk. However, primary healthcare has limited resources to work with modifying lifestyle habits. This is the first pilot study to test the feasibility of text message-based lifestyle intervention in patients with hypertension in Swedish primary healthcare. Whether significant improvement in cardiovascular risk factors may be achieved in a larger study population remains to be evaluated.

**Key points**
This pilot randomized controlled trial (RCT) is the first study to evaluate the feasibility of text message-based lifestyle advice to patients with hypertension in Swedish primary healthcare.
•All feasibility criteria for the pilot study were fulfilled. This outcome means that a larger study with a similar design may be conducted.
•The study was not powered to find significant changes in cardiovascular risk factors. Nevertheless, after six months we found favorable trends for all secondary outcomes in the intervention group compared to control.
•If a future larger study can show significant results, this intervention could serve as a useful tool in everyday primary healthcare.

## Introduction

The globalization of unhealthy lifestyles and demographic aging of the world’s population has contributed to the fact that high blood pressure (BP) is classified by the World Health Organization as the world’s leading risk for mortality [1]. Although most high-income countries have had favorable trends with decreasing prevalence of high BP, a global increased prevalence of raised BP has been shown in a recent large meta-analysis [2]. Hypertension is a key risk factor for cardiovascular diseases (CVD) [3]. Modifiable lifestyle risk factors associated with hypertension, including smoking, unhealthy diet and physical inactivity, account for approximately 80% of CVD [4]. The fact that 30–40% of individuals with hypertension have additional metabolic risk factors, such as dyslipidemia, insulin resistance and elevated blood-glucose [5], multiplies the risk for CVD [6]. In most cases, lifestyle intervention can reverse or reduce a patient’s unfavorable metabolic profile [7].

Primary healthcare centers in Sweden handle the majority of patients with hypertension. However, there are limited resources to support the lifestyle changes needed for primary and secondary prevention of CVD.

Interventions done using SMS (Short Message Services) have been shown to significantly improve compliance to medications, follow-up rate and disease monitoring [8–10]. A Swedish study, which used an interactive mobile phone intervention on BP, showed improved BP control by self-management of hypertension [11]. A randomized controlled trial (RCT) of CVD patients, who got weekly lifestyle focused SMS messages during six months, showed positive effects on BP, body mass index (BMI), physical activity and smoking cessation [12], as compared to the control group. Thus, communication by telehealth, e.g. SMS could constitute an additional tool to reach patients for lifestyle advice. SMS is a common, convenient and cheap method of communication that can reach a large proportion of a primary care population.

Although positive results have been indicated for text messaging interventions for CVD risk factors, lifestyle-promoting messages have not been evaluated as a treatment for hypertension in primary care. We conducted this pilot study to evaluate the feasibility of an RCT with SMS intervention to promote lifestyle changes in individuals with hypertension in primary healthcare. A secondary objective was to evaluate change on BP and other objective measures of cardiovascular risk and general health. A third objective was to evaluate the patients’ acceptability and utility of the SMS intervention.

## Materials and methods

### Trial design and participants

We conducted a three-center parallel-group RCT with a six month follow-up. A Case Report Form (CRF) was followed and controls were made by an external research monitor. The study was registered at ClinicalTrials.gov (NCT03442257).

In March–June 2018, patients aged 40–80 years with diagnosed hypertension were invited consecutively during doctor or nurse visits at three primary health care centers in southern Sweden. The three primary health care centers differed in socioeconomic status (according to predefined Care Need Index) [13] and healthcare burden (Adjusted Clinical Groups) [14]. Patients who were interested in participating were given written information about the study and were contacted within two weeks by a research assistant to provide further information. Those who were willing to participate were invited to the primary health care centers for the baseline visit, where a written informed consent was signed before the examination.

Patients were eligible if they fulfilled the following criteria: 40–80 years, had documented hypertension (defined by the International classification of disease ICD-10, diagnose code I10.9) and owned a mobile phone compatible with SMS. Exclusion criteria were: history of prior CVD (myocardial infarction, stroke, transient ischemic attack (TIA), intermittent claudication or abdominal aortic aneurysm) reported by recruiting physician or by the patient in the questionnaire; BP at baseline visit ≥180/110 mmHg or systolic BP <120 mmHg; serious illness with short life expectancy (<1 year); dementia/serious psychiatric disease or predicted inability to comply with the study protocol (e.g. language difficulties or interpreter needs).

### Randomization

Randomization was performed after completion of baseline assessments and questionnaires. A computer-generated random number schedule with block sizes of four was prepared. To ensure allocation concealment, a collaborator outside of the research project performed the randomization. Information about group affiliation was delivered to the patients by postal mail. The research assistant, the patients’ primary care physicians, as well as the researchers were blinded to group allocation. If the patients had questions or wanted to exit the study, they could call a telephone number and speak to a collaborator not involved in the data analysis.

### Intervention

The intervention consisted of regularly delivered SMS messages that aimed to remind, encourage and motivate patients to pursue healthy lifestyle changes. After baseline measurement, participants in the intervention group, in addition to their usual anti-hypertensive treatment, received four semi-personalized SMS messages per week for six months. Each week, the participants received one SMS with health information concerning each of the following groups: A. Physical activity, B. Tobacco use, C. Dietary habits, and D. Cardiovascular health in general, except for nonsmokers who, instead of the tobacco use-SMS, got one extra randomly selected SMS from group A, C or D (Table 1). The messages were sent at random times during daytime between 9 AM and 7 PM. The SMS messages were initially developed by the authors using lifestyle recommendations based on Swedish national guidelines [15–17], and edited by an expert group at the Centre for Lifestyle Habits in Malmö. This group included physiotherapists, dieticians and a physician specially trained in encouraging healthy lifestyle habits.

**Table 1.** Examples of text messages sent to the intervention group.

Category	Text message
Physical activity	‘Hi [NAME]. Regular physical activity improves both blood pressure and blood lipids. In addition, inflammation in the vessels may decrease, which contributes to the reduced risk of cardiovascular disease.’
	’Hi [NAME]. Reducing your sedentary time improves your health. Make a break by stretching your legs every 30 minutes when you are sedentary for a longer period.’
Tobacco use	‘Hi [NAME]. Did you know that your blood pressure and heart rate decrease already 20 minutes after smoking cessation?’
	‘Hi [NAME]. Instead of taking a smoking break, try to go for a brisk walk.’
Diet	‘Hi [NAME]. Wholemeal intake may reduce the risk for type 2 diabetes, cardiovascular disease and colorectal cancer.’
	‘Hi [NAME]. Too much salt intake may increase your blood pressure, which in turn increases the risk for myocardial infarction, heart failure, stroke and kidney failure.’
Cardiovascular health	‘Hi [NAME]. Home blood pressure monitoring with an upper arm cuff is a good complement to blood pressure measurement at the primary healthcare center.’
	‘Hi [NAME]. High blood pressure is the single most important treatable risk factor for cardiovascular disease, and that is why we strive to reach a normal blood pressure with medication.’

The control group received usual care.

### Measurements

Data were collected at baseline and after six months. BP was measured following the guidelines of the European Society of Hypertension [18], i.e. in the right arm in a sitting position after 5–10 min of rest with validated electronic blood pressure devices (Omron 705-IT, Omron Health Care Co., Kyoto, Japan). The mean of two readings was calculated (mean of three readings when the first and second readings differed by >5 mm Hg). Heart rate, weight, height, BMI, waist circumference, total cholesterol, high-density lipoprotein (HDL), low-density lipoprotein (LDL) and HbA1c were also measured at baseline and at six months. Furthermore, the patients completed a short questionnaire for evaluation of medical history, medication, tobacco use, self-rated health (SRH) and health-related quality of life. SRH was measured by a Likert scale *via* a simple question ‘How would you rate your general health?’ with five response options: very good, good, fair, poor or very poor [19]. Health-related quality of life was measured by the EQ5D-5L questionnaire, including the EQ visual analogue scale 0–100 (100 = the best health you can imagine) (EQ VAS) [20,21]. The EQ5D-5L questionnaire rates the level of impairment across five dimensions (mobility, self-care, usual activities, pain/discomfort, and anxiety/depression) and is measured on a five graded Likert scale. EQ-5D-5L index value was calculated through EQ-5D-5L Index Value Calculator developed by the EuroQol Group [22].

To evaluate the experience of the SMS messages, intervention participants received a separate questionnaire by postal mail 6–12 months after the intervention was completed. The questionnaire contained 13 statements about the acceptability and utility of the intervention and the participants answered by checking the most appropriate option on a five graded Likert scale (from disagree to strongly agree). The participants were also able to leave comments in free text and to give examples of SMS messages that they had favored or disliked.

### Outcomes

The primary outcome was feasibility of the study protocol, as defined prior to the study by the following three criteria [23]:

1. Recruitment rate. A recruitment rate of ≥55% was considered successful [12].
2. Dropout rate. A maximum of 15% dropout rate was considered acceptable, according to the power calculation for the full-scale study.
3. Eligibility criteria. The eligibility criteria were considered sufficient if 60 patients could be included from the three primary health care centers during the study period of four months.

If all three feasibility criteria were fulfilled, a larger study could be conducted without further changes in the protocol. If some of the criteria were not fulfilled, the protocol and design will be changed accordingly.

The secondary outcomes were: change in systolic and diastolic BP, BMI, waist circumference, total cholesterol, LDL, HDL, tobacco use, HbA1c, SRH, EQ5D-5L and EQ VAS. The acceptability and utility of the SMS intervention were evaluated through questionnaires.

### Statistical analysis

Data were analyzed using IBM SPSS Statistics 25 (IBM Corp., Released 2017, Armonk, NY, USA). For calculation of differences between groups; 2 tailed *t*-test was used for continuous variables, Mann–Whitney *U* test for categorical variables, and Pearson Chi-Square test for discrete variables. The EQ5D-5L index was calculated using EQ5D-5L index value for UK, through EQ-5D-5L Index Value Calculator developed by the EuroQol Group [22].

Mean differences in end points between intervention and control groups at six month follow up were calculated by ANCOVA, with baseline values used as covariates [24]. No other covariates were added, as the study sample was too small.

Prior to the study, we estimated that a total number of 60 patients would be sufficient to evaluate the feasibility of the intervention and the logistics of the assessments.

### Ethical considerations

Patients provided written informed consent at the baseline visit. The study was approved by the Regional Ethical Review Board in Lund, Sweden (Dnr: 2017/674). If extreme measures were found at baseline or follow-up visit, the patient’s primary care physician was informed. For the follow-up questionnaire, an additional ethical approvement was obtained (Dnr: 2019-01833) and a written consent form was provided by the participants.

## Results

**Figure 1.**
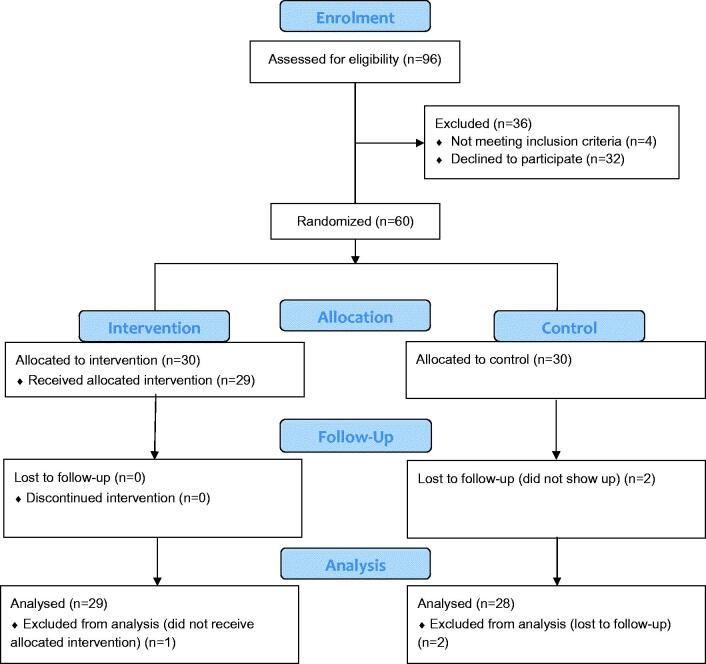
Enrollment of participants.

A total of 96 patients were invited to participate in the study *via* a simple question by the nurse or doctor during an ordinary visit at the primary health care center (Figure 1). One third (*n* = 32) declined. Reasons for declining participation were not registered at this point. At the baseline assessment, four patients did not meet the inclusion criteria (too low BP (*n* = 3), too high BP (*n* = 1)). Sixty patients were allocated to the study and two were lost to follow-up. Moreover, one participant in the intervention group did not receive the allocated intervention (information was never sent to the SMS company) and is therefore not included in the analysis. All 29 participants who received the SMS intervention answered the acceptability and utility questionnaire.

Mean age was 67.0 years and 57% were women, 82% were overweight (BMI ≥ 25 kg/m^2^, *n* = 49) and large waist circumference was found in 91% of the women (≥88 cm) and in 69% of the men (≥102cm). Mean BP was 141/79 mm Hg. There were no significant differences between the two groups at baseline. All participants except two were on antihypertensive medication (Table 2).

**Table 2.** Baseline characteristics of the intervention group and the control group.

	SMS group *n* = 30	Control group *n* = 30	*p* Value
Age, years	69.1 (5.4)	64.8 (10.5)	0.05
Female gender, *n* (%)	15 (50%)	19 (63%)	0.30
BMI, kg/m²	28.6 (5.1)	29.7 (4.5)	0.41
Waist circumference, cm	102.5 (14.6)	100.1 (12.5)	0.50
SBP, mmHg	141.7 (13.8)	141.1 (13.8)	0.86
DBP, mmHg	78.7 (8.9)	79.9 (9.5)	0.62
HbA1c, mmol/mol	39.4 (9.3)	38.5 (5.1)	0.64
Total cholesterol, mmol/l	5.1 (1.1)	5.2 (1.1)	0.70
LDL, mmol/l	3.3 (1.0)	3.4 (1.0)	0.60
HDL, mmol/l	1.6 (0.6)	1.5 (0.4)	0.49
Number of antihypertensive drugs	1.7 (0.9)	1.6 (0.7)	0.74
EQ5D-5L index	0.84 (0.15)	0.83 (0.13)	0.72
EQ VAS 0–100	88.5 (12.1)	83.2 (12.4)	0.10
SRH 1–5, median (SD)	2 (0.8)	2 (0.6)	0.07^§^

Means (SD) unless stated otherwise.

BMI: body mass index; DBP: diastolic blood pressure; SBP: systolic blood pressure; SRH: self-rated health.

2 tailed *t*-test for continuous variables, ^§^Mann–Whitney *U* test for categorical variables, Pearson Chi-Square test for discrete variables.

All three feasibility criteria were fulfilled: Recruitment rate ≥55% (60/96 = 63%); dropout rate <15% (2/60 = 3%) and 60 patients were included during the four-month inclusion period.

### Effect on BP and other CVD risk factors

Table 3 shows the follow-up measures (adjusted for baseline) and mean differences between the groups for all CVD risk factor outcome variables. There were no significant differences in mean change of any outcome variables between the SMS and control groups. However, all secondary outcome parameters in the SMS group showed favorable trends compared to the control group (Table 3). Three participants were smokers at baseline (SMS group *n* = 2, control group *n* = 1), none of these had quit smoking at follow-up.

**Table 3.** Outcome variables at six months follow up, adjusted for baseline values, and mean difference between SMS group and control group, One-way ANCOVA analysis.

	Adjusted mean (95 % CI)			
Parameter	SMS group (*n* = 29)	Control group (*n* = 28)	Mean difference: SMS-control (95 % CI)	*p* Value for difference
Systolic BP, mm Hg	140 (136–144)	144 (140–148)	−4 (−10 to 2)	0.19
Diastolic BP, mm Hg	78 (75–81)	79 (76–81)	−1 (−5 to 3)	0.70
BMI, kg/m^2^	28.7 (28.4–29.1)	29.1 (28.7–29.5)	−0.4 (−0.9 to 0.1)	0.13
Waist circumference, cm	100.5 (99.1–102.0)	102.1 (100.7–103.6)	−1.6 (−3.7 to 0.4)	0.12
HbA1c, mmol/mol	37.4 (35.1–39.6)	38.5 (36.2–40.8)	−1.1 (−4.3 to 2.1)	0.50
Total cholesterol, mmol/l	5.0 (4.8–5.1)	5.2 (5.0–5.4)	−0.2 (−0.5 to 0.1)	0.14
LDL, mmol/l	3.1 (2.9–3.3)	3.3 (3.1–3.5)	−0.2 (−0.4 to 0.1)	0.23
HDL, mmol/l	1.6 (1.5–1.6)	1.5 (1.4–1.6)	0.1 (−0.0 to 0.2)	0.10
EQ5D-5L index	0.8 (0.8–0.9)	0.8 (0.8–0.9)	0.01 (−0.04 to 0.05)	0.83
EQ VAS	88 (84–92)	82 (78–86)	6 (0 to 12)	0.06
SRH 1–5	1.9 (1.7–2.2)	2.0 (1.8–2.3)	−0.1 (−0.4 to 0.3)	0.70

BMI: body mass index; DBP: diastolic blood pressure; SBP: systolic blood pressure; SRH: self-rated health.

### Acceptability and utility of the intervention

All of the 29 patients who got the SMS intervention answered the acceptability and utility questionnaire. Seventy-six percent agreed (answered agree or strongly agree) that the SMS messages gave a reminder of healthy lifestyle habits, and 35% agreed that the SMS messages gave new knowledge (Supplementary Figures 1 and 2). More than one third (38%) stated that the SMS messages made them more physically active and one fourth (24%) had changed to healthier dietary habits. Ninety-seven percent read all the text messages for the first three months and about 93% also during the last three months. Half of the intervention group (52%) saved the text messages and 45% showed the messages to family and friends.

## Discussion

This pilot study showed that all three feasibility criteria were fulfilled. This outcome means that a future larger study can be conducted without any major changes being made to the study protocol. The study also showed that the intervention was accepted to a high degree by the participants. A favorable trend was seen for all pre-defined outcome variables which indicates that the lifestyle advice delivered *via* SMS may have contributed to the improvement. Hence, there is a need for larger RCTs to further evaluate the effects of this intervention on CVD risk factor variables.

Three quarters of the patients in the intervention group stated that the SMS messages gave a reminder of healthy lifestyle habits and 35% thought that the SMS messages gave them new knowledge (Supplementary Figures 1 and 2). Nowadays, the time for working with lifestyle modification in primary healthcare is very limited [25]. The commonly used methods are patient-centered with the aim of empowering patients to change lifestyle habits, but primary care personnel have reported a lack of skills in lifestyle counseling and experienced patients’ unwillingness to change lifestyle habits [26].

If a future larger study was to show significant improvement in lifestyle habits and CVD risk factors, our SMS tool may be implemented in everyday primary healthcare for patients with hypertension thus providing a complementary tool for lifestyle changes. The study was designed to observe the effect of a simple one-way SMS without an interactive approach, as we believe this could be used more widely in the clinical setting by not requiring additional personnel involvement. In addition, an earlier published three-armed RCT (control, simple text messages, interactive text messages) did not demonstrate any further effect on BP control in the group with interactive text messages [27].

In the acceptability and utility questionnaire, some of the participants asked for information that is more advanced, and for more web links to follow. This could perhaps enhance the impact of the intervention. However, the aim of this study was to create an intervention suitable and accessible for a wide range of subgroups of the population, e.g. people with simple mobile phones, elderly people not used to advanced mobile phone applications and people with different educational levels or technical skills. With this widespread approach, it is inevitable there will be different opinions on the intervention. Even though the recruitment criteria were fulfilled, the recruitment process took more time than expected. This was mainly due to lack of eligible patients because of the exclusion criterion of prior CVD. To be able to conduct a future larger study within a reasonable time, we plan to expand the inclusion criteria to patients with prior CVD. These patients may even be more aware and motivated to implement lifestyle changes, and a recent Australian study has shown positive results from SMS lifestyle intervention in patients with prior myocardial infarction [12].

Due to the nature of the intervention in this study, it was not possible to blind the participants [28]. We cannot rule out the effect on the patients just being in the intervention group in an RCT (performing bias). Vice versa applies to the patients in the control group, knowing they signed up for a study with text messages, and ending up in the control group, not receiving any messages. A solution to this could be a delayed intervention for the control group or cross-over design, but this would double the time for the study and was therefore not considered to be feasible in this study.

Three primary health care centers from different areas were chosen and they differed by both Adjusted Clinical Groups and Care Need Index. Thus, the study reflected a population with a range of disease burden and socioeconomic status. However, in this small pilot study, it is not possible to evaluate the generalizability to a larger population.

In conclusion, this pilot study met all feasibility criteria for a full-scale study and the participants were positive to a high extent regarding the SMS intervention. A full-scale study is needed to evaluate whether a significant improvement of cardiovascular risk factors may be achieved in a primary healthcare population.

## Supplementary Material

Supplemental Material
