# Supplementary material for: Text message-based lifestyle intervention in primary care patients with hypertension: a randomized controlled pilot trial
Source: Scand J Prim Health Care. 2020 Jul 20;38(3):300–7. doi: 10.1080/02813432.2020.1794392 (PMC7470149; doi:10.1080/02813432.2020.1794392)
Supplement: Supplemental Material [file IPRI_A_1794392_SM2929.docx]

**Supplementary Figure 1. Results from the follow up questionnaire to intervention participants, n=29. Five graded Likert scale 1=Strongly disagree 5=Strongly agree.**

**Supplementary Figure 2. Results from the follow up questionnaire to intervention participants, n=29. Five graded Likert scale 1=Strongly disagree 5=Strongly agree.**
